# International Conference on Women and Infectious Diseases: from Science to Action

**DOI:** 10.3201/eid0912.030870

**Published:** 2003-12

The International Conference on Women’s Health and Infectious Diseases, sponsored by the Centers for Disease Control and Prevention (CDC) and partners, will be held at the Marriott Marquis, Atlanta, Georgia, February 27–28, 2004. Intended for clinicians, scientists, women’s health advocates, health educators, public health workers, academicians, and representatives from all levels of government and from community-based, nonprofit, philanthropic, and international organizations, the conference will promote prevention and control of infectious diseases among women worldwide.

Featured sessions will include women and HIV/AIDS, perinatal infectious diseases, immunizations, links between infectious and chronic diseases, and the impact of globalization. Other topics include infectious disease disparities, gender-appropriate interventions, and effective health communication.

Speakers will include, Julie L. Gerberding, CDC director, who will speak about the impact of infectious diseases on women; Carol Bellamy, executive director, United Nations Children’s Fund (UNICEF), who will speak about globalization and its effect on infectious diseases among women; and Mirta Roses Periago, director, Pan American Health Organization (PAHO), will speak about prevention of infectious diseases among women globally.

For information, about cost and registration, contact the Office of Minority and Women’s Health, National Center for Infectious Diseases, CDC, at Web site: www.womenshealthconf.org; email: omwh@cdc.gov; or phone: BeJaye Roberts, 404-371-5311.

